# Predictive values of bilirubin for in-hospital adverse events in patients with ST-segment elevation myocardial infarction after primary percutaneous coronary intervention

**DOI:** 10.1016/j.clinsp.2023.100306

**Published:** 2023-11-08

**Authors:** Chen Ying, Cun-Fei Liu, De-Qun Guo, Zheng-Ren Du, Yan-Jin Wei

**Affiliations:** a Clinical Medical College, Weifang Medical University, Weifang City, Shandong Province, China; b Department of Cardiology, Linyi People's Hospital, Weifang Medical University, Linyi City, Shandong Province, China

**Keywords:** Bilirubin, ST-segment elevation myocardial infarction, Primary percutaneous coronary intervention, Major adverse cardiovascular events

## Abstract

**Purpose:**

To investigate the association between serum bilirubin levels and in-hospital Major Adverse Cardiac Events (MACE) in patients with ST-segment Elevation Myocardial Infarction (STEMI) undergoing primary Percutaneous Coronary Intervention (PCI).

**Methods:**

A total of 418 patients with STEMI who underwent primary PCI were enrolled from October 1^st^, 2021 to October 31^st^ 2022. The average age of enrolled participants was 59.23 years, and 328 patients (78.50%) were male patients. Patients were divided into MACE (patients with angina pectoris after infarction, recurrent myocardial infarction, acute heart failure, cardiogenic shock, malignant arrhythmias, or death after primary PCI) (n = 98) and non-MACE (n = 320) groups. Univariate and multivariate logistic regression analyses were performed to estimate the association between different bilirubin levels including Total Bilirubin (TB), Direct Bilirubin (DB), Indirect Bilirubin (IDB), and risk of in-hospital MACE. The area under the Receiver Operating Characteristic (ROC) curve was used to determine the accuracy of bilirubin levels in predicting in-hospital MACE.

**Results:**

The incidence of MACE in STEMI patients increased from the lowest to the highest bilirubin tertiles. Multivariate logistic regression analysis showed that increased total bilirubin level was an independent predictor of in-hospital MACE in patients with STEMI (p for trend = 0.02). Compared to the first TB group, the ORs for risk of MACE were 1.58 (95% CI 0.77‒3.26) and 2.28 (95% CI 1.13‒4.59) in the second and third TB groups, respectively. The ROC curve analysis showed that the areas under the curve for TB, DB and IDB in predicting in-hospital MACE were 0.642 (95% CI 0.578‒0.705, p < 0.001), 0.676 (95% CI 0.614‒0.738, p < 0.001), and 0.619 (95% CI 0.554‒0.683, p < 0.001), respectively.

**Conclusions:**

The current study showed that elevated TB, DB, and IDB levels are independent predictors of in-hospital MACE in patients with STEMI after primary PCI, and that DB has a better predictive value than TB and IDB.

## Highlight

• Adverse cardiac events after primary PCI are still common in STEMI patients.
• Elevated serum bilirubin levels are independent predictors of adverse cardiac events in STEMI patients.
• Direct bilirubin has a better predictive value for adverse cardiac events in STEMI patients.

## Introduction

ST-segment Elevation Myocardial Infarction (STEMI), a common and serious condition, is one of the leading causes of cardiac-related death.^1^^,^^2^ Primary Percutaneous Coronary Intervention (PCI) has become the preferred treatment for STEMI, achieving rapid vascular recanalization and patient survival.^3^ However, some patients remain prone to Major Adverse Cardiac Events (MACE) after primary PCI.^4^ Therefore, early identification of high-risk patients is important.

Under aerobic conditions, heme is catalyzed to bilirubin by heme oxygenase and bilirubin reductase.^5^ In clinical practice, bilirubin is commonly used as a biomarker of liver function and cholestasis. In recent years, several studies have investigated the correlation between bilirubin levels and cardiovascular diseases. Bilirubin can effectively inhibit low-density lipoprotein oxidation and scavenge superoxide and peroxide free radicals in vivo at physiological concentrations and may therefore have a beneficial effect against cardiovascular diseases.^6^^,^^7^ However, the conclusions between serum bilirubin levels with cardiovascular diseases in clinical studies showed an interesting paradox. Some studies have found that bilirubin levels are negatively correlated with the incidence and long-term prognosis of coronary heart disease and have a protective effect against cardiovascular diseases.^1^^,^^6^^,^^8^ Other studies found that the protective effect of serum bilirubin in patients with Acute Myocardial Infarction (AMI) is diminished or eliminated after adjusting for traditional cardiovascular risk factors.^9^, ^10^, ^11^ To date, few studies have focused on the relationship between bilirubin levels and the risk of in-hospital MACE in patients with STEMI after primary PCI, and the results have been inconsistent.^12^^,^^13^ Moreover, the association between in-hospital MACE with different bilirubin components including Total Bilirubin (TB), Direct Bilirubin (DB), and Indirect Bilirubin (IDB) is unclear. Therefore, the authors conducted the present study to explore the correlation between bilirubin levels and in-hospital MACE in patients with STEMI who underwent primary PCI.

## Methods

### Study populations

The authors prospectively enrolled 418 patients with STEMI who underwent primary PCI between October 2021 and October 2022. All patients were initially diagnosed with STEMI based on clinical guidelines.^14^ Patients with a history of heart failure, severe arrhythmia, severe valvular heart disease, previous myocardial infarction, coronary stent implantation or coronary bypass grafting, liver and kidney dysfunction, new infections, hematological disorders, or malignancies were excluded from the study. Written informed consent was obtained from all participants, and the study was approved by the Ethics Committee of Linyi People's Hospital.

The endpoints were MACE during hospitalization, including angina pectoris after infarction, recurrent myocardial infarction, acute heart failure, cardiogenic shock, malignant arrhythmias (including ventricular fibrillation, ventricular flutter, persistent ventricular tachycardia, third-degree atrioventricular block, second-degree type II atrioventricular block, and supraventricular tachycardia leading to hemodynamic disturbance), and death.

### Surgical data

All patients underwent primary PCI performed by two experienced coronary interventional experts after admission. Only the culprit vessels were treated during primary PCI. Surgical-related information, including Gensini score,^15^ surgical methods (coronary stent implantation or only percutaneous transluminal coronary angioplasty), culprit vessels, number of lesions (vessel stenosis > 70%), and number of implanted stents, was collected for further analysis.

### Baseline information and laboratory measurements

Venous blood samples were obtained from all subjects within 24h after primary PCI and sent to the laboratory for routine biochemical analysis (including hematologic parameters, liver and kidney function biomarkers, C-reactive protein, troponin-T, NT-proBNP, and blood lipids) according to standard methods. Transthoracic Doppler echocardiography (Vivid 7, GE Healthcare, USA) was performed on all patients during the first 24h. Baseline clinical data including height, weight, blood pressure, heart rate, smoking history, alcohol consumption history, hypertension, diabetes, and Killip classification were collected.

### Statistical analysis

Quantitative data conforming to a normal distribution were expressed as the means ± Standard Deviation (SD). The Student's *t*-test, one-way ANOVA, or Kruskal-Wallis H test was used for comparisons between two or multiple groups. Quantitative data with non-normal distributions were expressed as medians (25^th^ and 75^th^ percentiles), and non-parametric tests (Mann-Whitney or Kruskal-Wallis tests) were used for comparisons between two or multiple groups. Categorical data are expressed as numbers and percentages, and the Chi-Square test was used for comparisons between two or multiple groups. Univariate and multivariate binary logistic regression analyses were performed to evaluate the correlation between bilirubin levels and in-hospital MACE after primary PCI. The predictive value of bilirubin levels for in-hospital MACE was evaluated using a Receiver Operating Characteristic (ROC) curve. The Areas Under the Curve (AUC) for TB, DB and IDB were compared using the Delong method. IBM SPSS Statistics for Windows, version 26 (IBM Corp., Armonk, NY, USA) was used for the above analyses. Statistical significance was set at p-value < 0.05.

## Results

### Characteristics of patients between the MACE and non-MACE groups

In total, 418 patients with STEMI were included in this study. The baseline clinical characteristics of the patients are shown in Table 1. A total of 98 events was observed during hospitalization and the incidence of MACE was 23.4%. Overall, the patients in the MACE group were older (mean age: 62.94 vs. 58.1 years, p = 0.001), had longer hospitalization stays (11.86 vs. 8.44 days, p < 0.001), and had higher Killip classes (classes II–IV) (45% vs. 25.9%, p < 0.001). However, there were no significant differences in sex, history of smoking, alcohol consumption, hypertension, or diabetes (Table 1).

**Table 1:** Baseline characteristics of study patients.

	Total (n = 418)	Non-MACEs (n = 320)	MACEs (n = 98)	p-value
Age (years)	59.23 ± 12.98	58.10 ± 12.71	62.94 ± 13.24	0.001
Male (%)	328 (78.50%)	249 (77.80%)	79 (80.60%)	0.555
BMI (kg/m^2^)	25.73 ± 3.79	25.93 ± 3.80	25.10 ± 3.72	0.058
SBP (mmHg)	121.34 ± 18.18	122.78 ± 17.43	116.65 ± 19.80	0.003
Smoking (%)	209 (50.00%)	164 (51.30%)	45 (45.90%)	0.356
Drinking (%)	140 (33.50%)	111 (34.70%)	29 (29.60%)	0.350
Hypertension (%)	182 (43.50%)	134 (41.90%)	48 (49.00%)	0.215
Diabetes (%)	87 (20.80%)	67 (20.94%)	20 (20.40%)	0.910
Killip classes (%)				<0.001
I	291 (69.60%)	237 (74.10%)	54 (55.10%)	
II	107 (25.60%)	79 (24.70%)	28 (28.60%)	
III	5 (1.20%)	2 (0.60%)	3 (3.10%)	
IV	15 (3.60%)	2 (0.60%)	13 (13.30%)	
CRP (mg/L)	4.77 (3.18, 10.25)	4.20 (3.10, 9.00)	7.00 (3.10, 22.55)	<0.001
cTnT (ng/mL)	4.96 (2.37, 8.36)	4.48 (2.11, 7.57)	6.47 (3.41, 10.00)	<0.001
NT-proBNP (pg/mL)	755.60 (357.3, 1442.0)	679.50 (284.4, 1222.3)	1371 (529.4, 2862.8)	<0.001
TB (umoL/L)	14.34 ± 7.17	13.62 ± 6.75	16.67 ± 7.98	<0.001
DB (umoL/L)	5.24 ± 2.60	4.90 ± 2.31	6.34 ± 3.16	<0.001
IDB (umoL/L)	9.16 ± 5.07	8.76 ± 4.91	10.45 ± 5.37	0.004
ALT (U/L)	45.63 (30.60, 68.15)	41.55 (28.10,79.19)	67.15 (40.90, 95.90)	<0.001
Cr (umoL/L)	70.23 ± 19.89	67.78 ± 16.70	78.21 ± 26.45	<0.001
FBG (mmoL/L)	7.51 ± 2.77	7.13 ± 2.47	8.76 ± 3.32	<0.001
TG (mmoL/L)	1.29 (0.91, 1.83)	1.35 (0.93, 1.85)	1.21 (0.85, 1.58)	0.028
TC (mmoL/L)	4.76 ± 0.96	4.78 ± 1.00	4.70 ± 0.80	0.428
HDL-C (mmoL/L)	1.07 ± 0.27	1.05 ± 0.26	1.14 ± 0.30	0.005
LDL-C (mmoL/L)	3.11 ± 0.81	3.13 ± 0.83	3.05 ± 0.76	0.430
LVEF (%)	51.54 ± 7.35	53.77 ± 5.15	44.26 ± 8.65	<0.001

BMI, Body Mass Index; SBP, Systolic Blood Pressure; CRP, C-reactive Protein; cTnT, cardiac Troponin T; NT-proBNP, N-terminal pro-Brain Natriuretic Peptide; TB, Total Bilirubin; DB, Direct Bilirubin; IDB, Indirect Bilirubin; AST, Aspartate Aminotransferase; ALT, Alanine Aminotransferase; Cr, Creatinine; FBG, Fasting Blood Glucose; TG, Triglyceride; TC, Total Cholesterol; HDL-C, High-Density Lipoprotein Cholesterol; LDL-C, Low-Density Lipoprotein Cholesterol; LVEF, Left Ventricular Ejection Fraction.

As to the laboratory parameters, the MACE group had significantly higher levels of bilirubin, CRP, cardiac Troponin T (cTnT), NT-proBNP, Alanine Aminotransferase (ALT), Creatinine (Cr), and fasting blood glucose (all p < 0.001) and lower triglyceride levels (p = 0.028). There were no significant differences in total cholesterol and low-density lipoprotein cholesterol levels between the two groups. In addition, the Left Ventricular Ejection Fraction (LVEF) was significantly lower in the MACE group than in the non-MACE group (44.26% vs. 53.77%, p < 0.001).

The surgery-related data are presented in Table 2. Significantly higher Gensini scores, a greater number of multiple vessel lesions (≥ 2), and a higher proportion of left main artery and left anterior descending lesions were observed in the MACE group. No significant difference was observed in the number of stents implanted between the MACE and non-MACE groups.

**Table 2:** Surgical information of study patients.

	Total (n = 418)	Non-MACEs (n = 320)	MACEs (n = 98)	p-value
Gensini score	65.95 ± 28.29	61.39 ± 26.55	80.85 ± 28.80	<0.001
Surgical method (%)				0.808
PTCA	65 (15.60%)	49 (15.30%)	16 (16.30%)	
CSI	353 (84.40%)	271 (84.70%)	82 (83.70%)	
Culprit vessels (%)				<0.001
LM	2 (0.50%)	0 (0.00%)	2 (2.00%)	
LAD	203 (48.60%)	141 (44.00%)	62 (63.30%)	
LCX	59 (14.10%)	50 (15.60%)	9 (9.20%)	
RCA	154 (36.90%)	129 (40.30%)	25 (25.50%)	
No of lesions (vessel stenosis > 70%) (%)				<0.001
1	268 (64.10%)	219 (68.4%)	49 (50.00%)	
2	109 (26.10%)	79 (24.70%)	30 (30.60%)	
3	41 (9.80%)	22 (6.90%)	19 (19.40%)	
No of implanted stents (%)				0.203
0	65 (15.60%)	49 (15.30%)	16 (16.30%)	
1	238 (56.90%)	185 (57.80%)	53 (54.10%)	
2	98 (23.40%)	72 (22.50%)	26 (26.50%)	
3	14 (3.30%)	13 (4.10%)	1 (1.00%)	
4	3 (0.70%)	1 (0.30%)	2 (2.00%)	

PTCA, Percutaneous Transluminal Coronary Angioplasty; CSI, Coronary Stent Implantation; LM, Left Main Artery; LAD, Left Anterior Descending; LCX, Left Circumflex; RCA, Right Coronary Artery.

### Characteristics of patients according to bilirubin tertiles

The authors further assessed the characteristics of patients according to TB tertiles (Table 3) (comparisons according to DB and IDB are shown in Supplemental Materials, Tables 1‒4). The prevalence of in-hospital MACE in the TB tertile groups was 14%, 21.4%, and 34.5%, respectively, with a significant linear trend association (p < 0.001). There was also a significant difference in sex and CRP, NT-proBNP, ALT, and Cr levels among the TB groups (all p < 0.05). However, no substantial differences were found in age, smoking, drinking, glucose, or lipid profiles (Table 3).

**Table 3:** Characteristics of the study populations according to TB tertiles.

	Total bilirubin			p-value
	< 10.69 (n = 136)	10.69∼15.39 (n = 140)	≥15.39 (n = 142)	
Age (years)	57.96 ± 13.32	60.16 ± 12.78	59.54 ± 12.85	0.353
Male (%)	98 (72.10%)	108 (77.10%)	122 (85.90%)	0.017
BMI (kg/m^2^)	25.46 ± 3.68	25.59 ± 3.86	26.14 ± 3.82	0.286
SBP (mmHg)	119.90 ± 16.78	119.89 ± 16.87	124.16 ± 20.36	0.075
Smoking (%)	69 (50.70%)	67 (47.90%)	73 (51.40%)	0.819
Drinking (%)	42 (30.90%)	43(30.70%)	55 (38.70%)	0.266
Hypertension (%)	64 (47.10%)	58 (41.40%)	60 (42.30%)	0.596
Diabetes (%)	26 (19.10%)	29 (20.70%)	32 (22.50%)	0.781
Killip classes (%)				0.461
I	100 (73.50%)	94 (67.10%)	97 (68.30%)	
II	33 (24.30%)	38 (27.10%)	36 (25.40%)	
III	2 (1.50%)	1 (0.70%)	2 (1.40%)	
IV	1 (0.70%)	7 (5.00%)	7 (4.90%)	
CRP (mg/L)	4.05 (3.10, 7.50)	4.65 (3.10, 10.25)	6 (3.10, 17.43)	0.006
cTnT (ng/mL)	5.26 (2.12, 8.71)	4.90 (2.37, 8.52)	4.82 (2.44, 7.96)	0.851
NT-proBNP (pg/mL)	528.20 (171.20, 1052.50)	764.75 (361.80, 1384.50)	1055 (554.25, 1928.00)	<0.001
ALT (U/L)	40.15 (25.33, 61.28)	45.05 (30.73, 66.50)	52.15 (36.73, 73.58)	0.008
Cr (μmoL/L)	69.40 ± 22.30	67.89 ± 17.35	73.33 ± 19.53	0.060
FBG (mmoL/L)	7.41 ± 3.01	7.54 ± 2.80	7.58 ± 2.52	0.871
TG (mmoL/L)	1.25 (0.82, 1.74)	1.24 (0.92, 1.85)	1.37 (0.98, 1.85)	0.646
TC (mmoL/L)	4.76 ± 0.93	4.84 ± 1.04	4.69 ± 0.90	0.436
HDL-C (mmoL/L)	1.06 ± 0.27	1.09 ± 0.28	1.07 ± 0.27	0.694
LDL-C (mmoL/L)	3.08 ± 0.72	3.16 ± 0.88	3.09 ± 0.82	0.658
LVEF	52.68 ± 5.90	52.06 ± 6.91	49.92 ± 8.66	0.029
Surgical Data				
Gensini score	60.70 ± 26.68	68.54 ± 27.06	68.44 ± 30.39	0.030
Surgical method (%)				0.602
PTCA	18 (13.20%)	22 (15.70%)	25 (17.60%)	
CSI	118 (86.80%)	118 (84.30%)	117 (82.40%)	
Culprit vessels (%)				0.005
LM	0	1 (0.70%)	1 (0.70%)	
LAD	53 (39.00%)	66 (47.10%)	84 (59.20%)	
LCX	25 (18.40%)	13 (9.30%)	21 (14.80%)	
RCA	58 (42.60%)	60 (42.90%)	36 (25.40%)	
No of lesions (vessel stenosis > 70%) (%)				0.040
1	92 (67.60%)	83 (59.30%)	93 (65.50%)	
2	39 (28.70%)	37 (26.40%)	33 (23.30%)	
3	5 (3.70%)	20 (14.30%)	16 (11.30%)	
No of implanted stents (%)				0.318
0	18 (13.20%)	22 (15.70%)	25 (17.60%)	
1	82 (60.30%)	71 (50.70%)	85 (35.70%)	
2	31 (22.80%)	38 (27.10%)	29 (20.40%)	
3	3 (2.20%)	8 (5.70%)	3 (2.10%)	
4	2 (1.50%)	1 (0.70%)	0	
MACEs (%)	19 (14.00%)	30 (21.40%)	49 (34.50%)	<0.001

BMI, Body Mass Index; SBP, Systolic Blood Pressure; CRP, C-Reactive Protein; cTnT, Cardiac Troponin T; NT-proBNP, N-terminal pro-Brain Natriuretic Peptide; TB, Total Bilirubin; DB, Direct Bilirubin; IDB, Indirect Bilirubin; AST, Aspartate Aminotransferase; ALT, Alanine Aminotransferase; Cr, Creatinine; TG, Triglyceride; TC, Total Cholesterol; HDL-C, High-Density Lipoprotein Cholesterol; LDL-C, Low-Density Lipoprotein Cholesterol; LVEF, Left Ventricular Ejection Fraction; PTCA, Percutaneous Transluminal Coronary Angioplasty; CSI, Coronary Stent Implantation; LM, Left Main Artery; LAD, Left Anterior Descending; LCX, Left Circumflex; RCA, Right Coronary Artery.

### Correlation of bilirubin with the risk of in-hospital MACE after primary PCI

Univariate and multivariate logistic regression analyses were performed to assess the association between bilirubin level and the risk of in-hospital MACE after primary PCI in patients with STEMI. The detailed results of the analyses are presented in Table 4. Compared to the first TB group, there was an increased unadjusted OR in the MACE group from 1.68 (95% CI 0.9‒3.16) in the intermediate TB group to 3.24 (95% CI 1.79‒5.89) in the third TB group (p for trend < 0.001). In the multifactorial regression analysis, serum TB remained an influential risk factor for in-hospital MACE after adjusting for several covariates including age, sex, hypertension, diabetes, CRP, culprit vessels, ALT, and Cr. The adjusted ORs of the incidence were 1.58 (95% CI 0.77‒3.26) and 2.28 (95% CI 1.13‒4.59) in the intermediate TB group and the third TB groups, respectively. Direct and indirect bilirubin levels with the risk of in-hospital MACE were also evaluated and are presented in the Supplemental Materials.

**Table 4:** Odds ratios (95% CIs) of MACEs according to tertiles of bilirubin.

		Q1	Q2	Q3	p for trend
**TB**	Model 1	1	1.68 (0.90, 3.16)	3.24 (1.79, 5.89)	<0.001
	Model 2	1	1.56 (0.82, 2.96)	3.06 (1.66, 5.62)	<0.001
	Model 3	1	1.58 (0.77, 3.26)	2.28 (1.13, 4.59)	0.020
**DB**	Model 1	1	1.59 (0.83, 3.05)	4.06 (2.23, 7.41)	<0.001
	Model 2	1	1.45 (0.75,2.80)	3.64 (1.96, 6.78)	<0.001
	Model 3	1	1.23 (0.60, 2.55)	2.42 (1.20, 4.88)	0.009
**IDB**	Model 1	1	1.64 (0.89, 3.02)	2.52 (1.40, 4.51)	0.002
	Model 2	1	1.64 (0.88, 3.05)	2.48 (1.36, 4.58)	0.003
	Model 3	1	1.88 (0.91, 3.89)	2.26 (1.12, 4.54)	0.026

Model 1: Unadjusted OR.

Model 2: Adjusted for sex and age.

Model 3: Adjusted for sex, age, hypertension, diabetes, CRP, culprit vessels, ALT, creatinine.

### Accuracy of bilirubin in predicting in-hospital MACE after primary PCI

**Figure 1:**
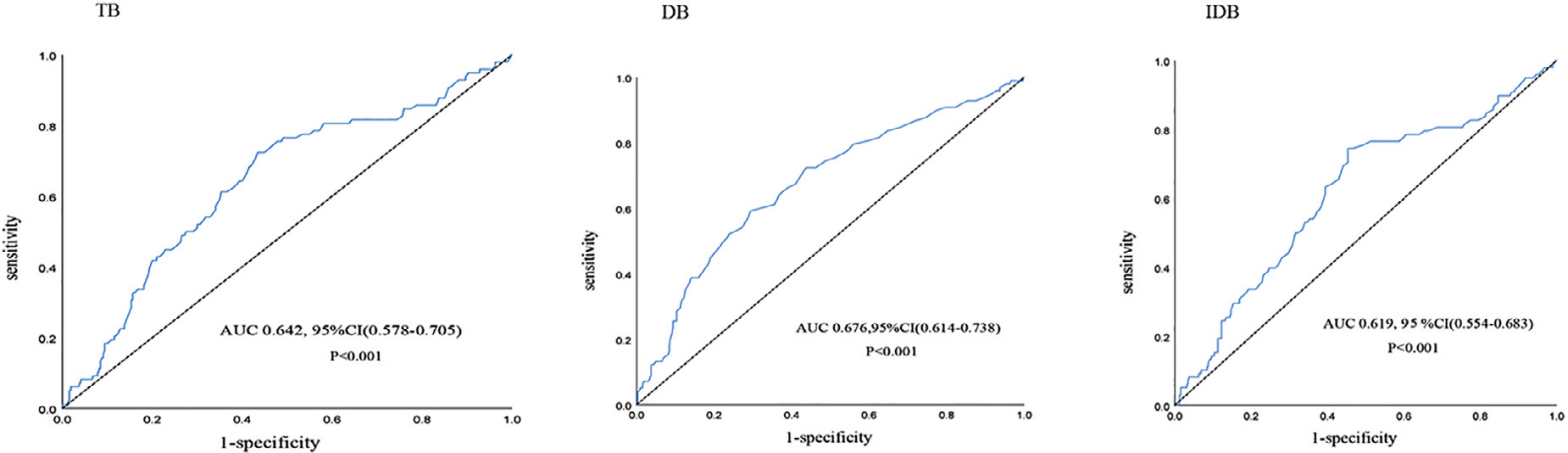
Predictive value of bilirubin for in-hospital MACEs.

The ROC curve was used to assess the predictive value of bilirubin levels for in-hospital MACE after primary PCI (Fig. 1). The optimal cut-off level for TB in predicting in-hospital MACE was 12.9 umoL/L, with a sensitivity of 72.4% and a specificity of 56.6% (AUC = 0.642, 95% CI 0.578‒0.705, p < 0.001). Meanwhile, the optimal cut-off level for DB was 5.35 umoL/L, with a sensitivity of 59.2% and specificity of 70.6% (AUC = 0.676, 95% CI 0.614‒0.738, p < 0.001), and the optimal cut-off level for IDB was 7.75 umoL/L, with a sensitivity of 74.5% and specificity of 54.7% (AUC = 0.619, 95% CI: 0.554-0.683, p < 0.001). In addition, the authors compared the differences between the AUCs for TB, DB, and IDB using the Delong method (all p < 0.001). Overall, the DB was slightly better than TB and IDB in predicting in-hospital MACE.

## Discussion

The main finding of this study was that serum bilirubin levels were independently associated with in-hospital MACE in patients with STEMI after primary PCI. Elevated serum bilirubin level is a simple and powerful predictor of in-hospital MACE in patients with STEMI treated with primary PCI.

In the presence of Heme Oxygenase and biliverdin reductase, heme is broken down into bilirubin. Bilirubin is produced by the mononuclear phagocytic system and transported into plasma where it is called indirect bilirubin. Free indirect bilirubin in plasma is bound to albumin and transported to hepatocytes, where it is converted to direct bilirubin by UDP glucuronosyltransferase. The antioxidative effects of bilirubin were first reported in 1954.^6^ Over the past few decades, an increasing number of studies have focused on the association between bilirubin levels and coronary heart disease. Basic and epidemiological studies have shown that excessive bilirubin is harmful. However, appropriate levels of bilirubin have anti-inflammatory, antioxidant, and antiproliferative effects that can modulate atherosclerosis and reduce the incidence of coronary heart disease.^16^, ^17^, ^18^, ^19^

However, controversy has arisen regarding the relationship between serum bilirubin levels and prognosis in patients with AMI. For example, Acet et al. showed that higher serum bilirubin levels were associated with increased MACE and death in patients with STEMI.^13^ Gul et al. found that high serum bilirubin levels were independent predictors of in-hospital cardiac mortality in patients with STEMI.^11^ However, Kim et al. demonstrated that low serum TB level is an independent predictor of long-term mortality.^20^ Another retrospective study found that lower serum bilirubin levels were positively associated with MACE in patients with AMI.^12^ The present study explored the association between bilirubin levels and in-hospital MACE in patients with STEMI who underwent primary PCI. The present study showed that serum bilirubin levels were independently and positively associated with in-hospital MACE in patients with STEMI after primary PCI. Compared with the first tertile, the incidence of MACE was significantly higher in the third tertile TB group (34.5% vs. 14%). The adjusted OR was 2.28 (95% CI 1.13‒4.59) even after adjustment for several covariates (age, sex, hypertension, diabetes, CRP, ALT, creatinine, and culprit vessels).

Severe oxidative stress reactions occur during STEMI and subsequent revascularization after primary PCI.^21^, ^22^, ^23^, ^24^ Bilirubin levels rise rapidly through stress-activated HO-1 activity to scavenge the increased levels of reactive oxygen radicals and exert anti-inflammatory and antioxidant effects. However, short-term sharp increases in bilirubin, which exerts anti-inflammatory and antioxidant effects to protect the myocardium, are unable to compensate for the overwhelming detrimental effects of myocardial infarction. Tuxun et al. concluded that the more severe the atherosclerosis, the higher the HO-1 enzyme activity and the more pronounced the increase in bilirubin levels after AMI.^25^ The present study showed that high serum bilirubin levels were significantly associated with increased severity of coronary artery lesions according to the Gensini score. Therefore, elevated bilirubin levels in patients with STEMI may be useful for predicting the short-term prognosis.

The study has several limitations. First, this was a single-center observational study and could only analyze the association between bilirubin levels and MACE. Secondly, the sample size was small. Finally, the authors only analyzed in-hospital patient outcomes; this study could have provided patients’ long-term outcomes and bilirubin levels with continued follow-up after discharge.

## Conclusion

In conclusion, the present study showed that serum bilirubin levels were positively associated with the incidence of in-hospital MACE in patients with STEMI after primary PCI. The present study suggests that elevated bilirubin levels are an independent predictor of in-hospital MACE in patients with STEMI and that DB has a better predictive value than TB and IDB levels.

## Authors’ contributions

Conceived and designed the experiments: Chen Ying, Cun-Fei Liu, Yan-Jin Wei. Analyzed the data: Chen-Ying, Cun-Fei Liu, De-Qun Guo, Zheng-Ren Du, Yan-Jin Wei. Contributed reagents/materials/analysis tools: Chen-Ying, Cun-Fei Liu, De-Qun Guo, Zheng-Ren Du, Yan-Jin Wei. Roles/Writing - original draft: ChenYing; Writing - review & editing: Cun-Fei Liu and Yan-jin Wei.

## Funding statement

The study was supported by a grant from the Science and Technology Development Plan of Linyi City (no 201919013) and a grant from the Natural Science Foundation of Shandong province (no ZR2020MH018).

## Conflicts of interest

The authors declare no conflicts of interest.

## Appendix Supplementary materials

Image, application 1

## References

[1] Shen H, Zeng C, Wu X, Liu S, Chen X. (2019). Prognostic value of total bilirubin in patients with acute myocardial infarction. Medicine.

[2] Frikha Z, Ferreira JP, Bozec E, McMurray J, Pitt B, Dickstein K (2018). Relation of high serum bilirubin to short-term mortality following a myocardial infarction complicated by left ventricular systolic dysfunction (from the high-risk myocardial infarction database initiative). Am J Cardiol.

[3] Anderson H, Masri SC, Abdallah MS, Chang AM, Cohen MG, Elgendy IY (2022). 2022 ACC/AHA key data elements and definitions for chest pain and acute myocardial infarction: a report of the American Heart Association/American College of Cardiology Joint Committee on Clinical Data Standards. J Am Coll Cardiol.

[4] Song L, Zhao X, Chen R, Li J, Zhou J, Liu C (2022). Association of PCSK9 with inflammation and platelet activation markers and recurrent cardiovascular risks in STEMI patients undergoing primary PCI with or without diabetes. Cardiovasc Diabetol.

[5] Li C, Wu W, Song Y, Xu S, Wu X. (2022). The nonlinear relationship between total bilirubin and coronary heart disease: a dose-response meta-analysis. Front Cardiovasc Med.

[6] Vítek L. (2012). The role of bilirubin in diabetes, metabolic syndrome, and cardiovascular diseases. Front Pharmacol.

[7] Hulea SA, Wasowicz E, Kummerow FA. (1995). Inhibition of metal-catalyzed oxidation of low-density lipoprotein by free and albumin-bound bilirubin. Biochim Biophys Acta.

[8] Li Y, Li DB, Zhao LD, Lv QB, Wang Y, Ren YF (2022). Effects of bilirubin on perioperative myocardial infarction and its long-term prognosis in patients undergoing percutaneous coronary intervention. World J Clin Cases.

[9] Jørgensen ME, Torp-Pedersen C, Finer N, Caterson I, James WP, Legler UF (2014). Association between serum bilirubin and cardiovascular disease in an overweight high-risk population from the SCOUT trial. Nutr Metab Cardiovasc.

[10] Mahabadi AA, Lehmann N, Möhlenkamp S, Kälsch H, Bauer M, Schulz R (2014). Association of bilirubin with coronary artery calcification and cardiovascular events in the general population without known liver disease: the Heinz Nixdorf Recall study. Clin Res Cardiol.

[11] Gul M, Uyarel H, Ergelen M, Akgul O, Karaca G, Turen S (2013). Prognostic value of total bilirubin in patients with ST-segment elevation acute myocardial infarction undergoing primary coronary intervention. Am J Cardiol.

[12] Wei S, Mao L, Liu B, Zhong L. (2014). Serum biomarkers and the prognosis of AMI patients. Herz.

[13] Acet H, Ertş F, Akıl MA, Polat N, Aydın M, Akyüz A (2014). A novel predictor of infarct-related artery patency before percutaneous intervention and in-hospital outcomes for ST-segment elevation myocardial infarction patients: serum bilirubin level. Postep Kardiol Inter.

[14] Thygesen K, Alpert JS, Jaffe AS, Chaitman BR, Bax JJ, Morrow DA (2018). Fourth universal definition of myocardial infarction (2018). J Am Coll Cardiol.

[15] Gensini GG. (1983). A more meaningful scoring system for determining the severity of coronary heart disease. Am J Cardiol.

[16] Celik T, Kaya MG, Akpek M, Yarlioglues M, Sarli B, Topsakal R (2014). Does Serum Bilirubin level on admission predict TIMI flow grade and in-hospital MACE in patients with STEMI undergoing primary PCI. Angiology.

[17] Lai X, Fang Q, Yang L, Chen X, Wang H, Ma L (2018). Direct, indirect and total bilirubin and risk of incident coronary heart disease in the Dongfeng-Tongji cohort. Ann Med.

[18] Lin JP, O'Donnell CJ, Schwaiger JP, Cupples LA, Lingenhel A, Hunt SC (2006). Association between the UGT1A1*28 allele, bilirubin levels, and coronary heart disease in the Framingham Heart Study. Circulation.

[19] Yang Y, Wang J, Wai SDA, Xu Y, Jiang H, Ma K (2022). Serum total bilirubin and long-term prognosis of patients with new-onset non-ST elevation myocardial infarction: a cohort study. Bmc Cardiovasc Disor.

[20] Kim HW, Choi DH, Lim L, Lee YM, Kang JT, Chae SS (2015). Usefulness of serum bilirubin levels as a biomarker for long-term clinical outcomes after percutaneous coronary intervention. Heart Vessels.

[21] Yayla Ç, Gayretli YK, Açar B, Unal S, Ertem AG, Akboga MK (2017). White blood cell subtypes and ratios in cardiovascular disease. Angiology.

[22] Serdar Z, Serdar A, Altin A, Eryilmaz U, Albayrak S (2007). The relation between oxidant and antioxidant parameters and severity of acute coronary syndromes. Acta Cardiol.

[23] Turan T, Menteşe Ü, Ağaç MT, Akyüz AR, Kul S, Aykan AÇ (2015). The relation between intensity and complexity of coronary artery lesion and oxidative stress in patients with acute coronary syndrome. Anatol J Cardiol.

[24] Tang C, Qian H, Wang D, Qiao Y, Yan G (2019). Prognostic value of serum total bilirubin after percutaneous coronary intervention in patients with acute coronary syndrome. Biomed Res Int.

[25] Tuxun M, Zhao Q, Xiang Y, Liu F, Shan CF, Zhou XR (2020). Predicting value of white cell count and total bilirubin on clinical outcomes in patients with ST-elevation myocardial infarction following percutaneous coronary intervention: a cohort study. BMJ Open.

