# Supplementary material for: Predictive values of bilirubin for in-hospital adverse events in patients with ST-segment elevation myocardial infarction after primary percutaneous coronary intervention
Source: Clinics (Sao Paulo). 2023 Nov 8;78:100306. doi: 10.1016/j.clinsp.2023.100306 (PMC10661652; doi:10.1016/j.clinsp.2023.100306)
Supplement: Supplementary file 1 [file mmc1.docx]

**CLINICS-D-23-00345_Supplementary Material**

**Supplemental Table 1** Characteristics of the study populations according to DB tertiles.

| **Variable** | **Direct bilirubin** | | | **p-value** |
| --- | --- | --- | --- | --- |
|  | **< 3.96**  **(n = 138)** | **3.96~5.52**  **(n = 140)** | **≥ 5.53**  **(n = 140)** |  |
| Age (years) | 56.77 ± 13.91 | 60.2 ± 12.09 | 60.69 ± 12.63 | 0.023 |
| Male (%) | 97 (70.3%) | 108 (77.1%) | 123 (87.9%) | 0.002 |
| BMI (kg/m^2^) | 25.52 ± 4.01 | 25.58 ± 3.47 | 26.1 ± 3.87 | 0.377 |
| SBP (mmHg) | 120.04 ± 16.36 | 121.04 ± 17.69 | 122.93 ± 20.25 | 0.406 |
| Smoking (%) | 68 (49.3%) | 67 (47.9%) | 74 (52.9%) | 0.69 |
| Drinking (%) | 43 (31.2%) | 38 (27.1%) | 59 (42.1%) | 0.023 |
| Hypertension (%) | 64 (46.4%) | 57 (40.7%) | 61 (43.6%) | 0.636 |
| Diabetes (%) | 26 (18.8%) | 33 (23.6%) | 28 (20%) | 0.598 |
| Killip classes (%) |  |  |  | 0.235 |
| I | 103 (74.6%) | 91 (65%) | 97 (69.3%) |  |
| II | 32 (23.2%) | 42 (30%) | 33 (23.6%) |  |
| III | 2 (1.4%) | 1 (0.7%) | 2 (1.4%) |  |
| IV | 1 (0.7%) | 6 (4.3%) | 8 (5.7%) |  |
| CRP (mg/L) | 4.45 (3.1, 8.28) | 4.5 (3.1, 9.5) | 5.85 (3.1, 20.25) | 0.024 |
| cTnT (ng/mL) | 4.76 (1.8, 8.35) | 5.47 (2.39, 9.12) | 4.91 (2.67, 7.97) | 0.543 |
| NT-proBNP (pg/mL) | 501.45 (182.08‒1045.5) | 791.65 (373.25, 1430) | 1055 (541.88, 1918.75) | <0.001 |
| FBG (mmoL/L) | 7.28 ± 2.63 | 7.51 ± 2.92 | 7.74 ± 2.77 | 0.389 |
| ALT (U/L) | 39.8 (25.08, 58.58) | 44.1 (30.95, 68.05) | 54.05 (37.65, 75.55) | <0.001 |
| Cr (umoL/L) | 67.12 ± 17.59 | 70.62 ± 22.51 | 72.91 ± 18.92 | 0.05 |
| TG (mmoL/L) | 1.3 (0.86, 1.85) | 1.26 (0.93, 1.85) | 1.3 (0.93, 1.78) | 0.917 |
| TC (mmoL/L) | 4.94 ± 1.01 | 4.73 ± 0.94 | 4.62 ± 0.9 | 0.021 |
| HDL-C (mmoL/L) | 1.05 ± 0.27 | 1.05 ± 0.27 | 1.12 ± 0.27 | 0.078 |
| LDL-C (mmoL/L) | 3.2 ± 0.8 | 3.12 ± 0.81 | 3.01 ± 0.81 | 0.154 |
| LVEF | 52.72 ± 6.25 | 52.36 ± 6.32 | 49.55 ± 8.8 | 0.004 |

BMI, Body Mass Index; SBP, Systolic Blood Pressure; CRP, C-Reactive Protein; cTnT, Cardiac Troponin T; NT-proBNP, N-terminal pro-Brain Natriuretic Peptide; TB, Total Bilirubin; DB, Direct Bilirubin; IDB, Indirect Bilirubin; FBG, Fasting Blood Glucose; ALT, Alanine Aminotransferase; Cr, Creatinine; TG, Triglyceride; TC, Total Cholesterol; HDL-C, High-Density Lipoprotein Cholesterol; LDL-C, Low-Density Lipoprotein Cholesterol; LVEF, Left Ventricular Ejection Fraction.

**Supplemental Table 2** Characteristics of the study populations according to IDB tertiles.

| **Variable** | **Indirect bilirubin** | | | **p-value** |
| --- | --- | --- | --- | --- |
|  | **< 6.5 (n =136)** | **6.5~9.79 (n = 139)** | **≥ 9.8 (n = 143)** |  |
| Age (years) | 59.2 ± 13.84 | 59.19 ± 12.72 | 59.31 ± 12.48 | 0.996 |
| Male (%) | 98 (72.1%) | 108 (77.7%) | 122 (85.3%) | 0.026 |
| BMI (kg/m^2^) | 25.41 ± 3.73 | 25.47 ± 3.9 | 26.3 ± 3.7 | 0.085 |
| SBP (mmHg) | 119.59 ± 16.96 | 118.91 ± 16.42 | 125.38 ± 20.23 | 0.004 |
| Smoking (%) | 68 (50%) | 67 (48.2%) | 74 (51.7%) | 0.837 |
| Drinking (%) | 41 (30.1%) | 43 (30.9%) | 56 (39.2%) | 0.207 |
| Hypertension (%) | 63 (46.3%) | 60 (43.2%) | 59 (41.3%) | 0.691 |
| Diabetes (%) | 27 (19.9%) | 27 (19.4%) | 33 (23.1%) | 0.711 |
| Killip classes (%) |  |  |  | 0.141 |
| I | 100 (73.5%) | 94 (67.6%) | 97 (67.8%) |  |
| II | 33 (24.3%) | 36 (25.9%) | 38 (26.6%) |  |
| III | 2 (1.5%) | 0 | 3 (2.1%) |  |
| IV | 1 (0.7%) | 9 (6.5%) | 5 (3.5%) |  |
| CRP (mg/L) | 4.2 (3.1, 7.78) | 4.5 (3.1, 10.1) | 6 (3.1, 16.8) | 0.096 |
| cTnT (ng/mL) | 5.35 (2.12, 8.71) | 4.73 (2.36, 8.31) | 4.97 (2.44, 7.97) | 0.86 |
| NT-proBNP (pg/mL) | 558.25 (180.43, 1232) | 747.5 (308.1, 1354) | 1063 (560.4, 1764) | 0.001 |
| [FBG(](javascript:;)mmol/L) | 7.47 ± 3.02 | 7.53 ± 2.73 | 7.53 ± 25.9 | 0.981 |
| ALT (U/L) | 40.15 (26.38, 61.95) | 45.9 (29.7, 68.1) | 49.4 (34.7, 70) | 0.21 |
| Cr (μmoL/L) | 70.48 ± 22.7 | 66.73 ± 15.97 | 73.41 ± 20.05 | 0.018 |
| TG (mmoL/L) | 1.15 (0.81, 1.69) | 1.3 (0.96, 1.85) | 1.36 (0.98, 1.85) | 0.899 |
| TC (mmoL/L) | 4.65 ± 0.95 | 4.9 ± 0.97 | 4.74 ± 0.95 | 0.101 |
| HDL-C (mmoL/L) | 1.06 ± 0.28 | 1.09 ± 0.27 | 1.07 ± 0.27 | 0.695 |
| LDL-C (mmoL/L) | 2.97 ± 0.73 | 3.24 ± 0.85 | 3.12 ± 0.82 | 0.025 |
| LVEF | 52.53 ± 6.19 | 51.91 ± 6.86 | 50.23 ± 8.58 | 0.094 |

**Supplemental Table 3** The surgical related data according to DB tertiles.

|  | **Direct bilirubin** | | | **p-value** |
| --- | --- | --- | --- | --- |
|  | **< 3.96 (n = 138)** | **3.96~5.52 (n = 140)** | **≥ 5.53 (n = 140)** |  |
| Surgical Data |  |  |  |  |
| Gensini score | 59.53 ± 26.26 | 68.5 ± 27.95 | 69.73 ± 29.63 | 0.004 |
| Surgical method (%) |  |  |  | 0.798 |
| Only PTCA | 21 (15.2%) | 20 (14.3%) | 24 (17.1%) |  |
| CSI | 117 (84.8%) | 120 (85.7%) | 116 (82.9%) |  |
| Culprit vessels (%) |  |  |  | 0.043 |
| LM | 0 | 1 (0.7%) | 1 (0.7%) |  |
| LAD | 55 (39.9%) | 65 (46.4%) | 83 (59.3%) |  |
| LCX | 25 (18.1%) | 18 (12.9%) | 16 (11.4%) |  |
| RCA | 58 (42%) | 56 (40%) | 40 (28.6%) |  |
| NO of lesions (vessel stenosis >70%) |  |  |  | 0.029 |
| 1 | 96 (69.6%) | 82 (58.6%) | 90 (64.3%) |  |
| 2 | 37 (26.8%) | 37 (26.4%) | 35 (25%) |  |
| 3 | 5 (3.6%) | 21 (15%) | 15 (10.7%) |  |
| NO of implanted stents (%) |  |  |  | 0.503 |
| 0 | 21 (15.2%) | 20 (14.3%) | 24 (17.1%) |  |
| 1 | 80 (58%) | 74 (52.9%) | 84 (60%) |  |
| 2 | 33 (23.9%) | 36 (25.7%) | 29 (20.7%) |  |
| 3 | 3 (2.2%) | 8 (5.7%) | 3 (2.1%) |  |
| 4 | 1 (0.7%) | 2 (1.4%) | 0 |  |

**Supplemental Table 4** The surgical related data according to IDB tertiles.

|  | **Indirect bilirubin** | | | **p-value** |
| --- | --- | --- | --- | --- |
|  | **< 6.5**  **(n = 136)** | **6.5~9.79**  **(n = 139)** | **≥ 9.8**  **(n = 143)** |  |
| Gensini score | 60.44 ± 27.41 | 67.29 ± 26.76 | 69.9 ± 29.88 | 0.016 |
| Surgical method (%) |  |  |  | 0.662 |
| Only PTCA | 18 (13.2%) | 23 (16.5%) | 24 (16.8%) |  |
| CSI | 118 (86.8%) | 116 (83.5%) | 119 (83.2%) |  |
| Culprit vessels (%) |  |  |  | 0.004 |
| LM | 1(0.7%) | 0 | 1 (0.7%) |  |
| LAD | 50 (36.8%) | 69 (49.6%) | 84 (58.7%) |  |
| LCX | 26 (19.1%) | 13 (9.4%) | 20 (14%) |  |
| RCA | 59 (43.4%) | 57 (41%) | 38 (26.6%) |  |
| NO of lesions (vessel stenosis >70%) (%) |  |  |  | 0.14 |
| 1 | 92 (67.6%) | 87 (62.6%) | 89 (62.2%) |  |
| 2 | 38 (27.9%) | 36 (25.9%) | 35 (24.5%) |  |
| 3 | 6 (4.4%) | 16 (11.5%) | 19 (13.3%) |  |
| NO of implanted stents (%) |  |  |  | 0.761 |
| 0 | 18 (13.2%) | 23 (16.5%) | 24 (16.8%) |  |
| 1 | 81 (59.6%) | 73 (52.5%) | 84 (58.7%) |  |
| 2 | 33 (24.3%) | 35 (25.2%) | 30 (21%) |  |
| 3 | 3 (2.2%) | 6 (4.3%) | 5 (3.5%) |  |
| 4 | 1 (0.7%) | 2 (1.4%) | 0 |  |
